# Supplementary figures and images for: A genomic snapshot of Salmonella enterica serovar Typhi in Colombia
Source: PLoS Negl Trop Dis. 2021 Sep 16;15(9):e0009755. doi: 10.1371/journal.pntd.0009755 (PMC8478212; doi:10.1371/journal.pntd.0009755)

## Slide 1
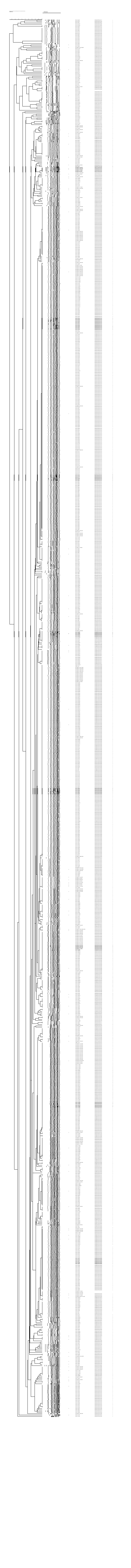

Supplement: S1 Fig — PFGE-XbaI dendrogram generated with Dice coefficient and UPGMA clustering method (tolerance and optimization 1,5%) of 1,077 isolates. The isolates showed 51.45% genetic similarity and represent 211 unique XbaI digestion patterns (as of June 2021). The grey dots indicate the isolates selected for WGS. (PPTX) [file pntd.0009755.s002.pptx]
