# Supplementary material for: A genomic snapshot of Salmonella enterica serovar Typhi in Colombia
Source: PLoS Negl Trop Dis. 2021 Sep 16;15(9):e0009755. doi: 10.1371/journal.pntd.0009755 (PMC8478212; doi:10.1371/journal.pntd.0009755)
Supplement: Attachment — Submitted filename: Cover letter20210720.docx [file pntd.0009755.s003.docx]

Professor Stephen Baker

Sgb47@medschl.cam.ac.uk

20^th^ July 2021

Re: Re-submission to PLoS Neglected Tropical Diseases

Dear editor,

I wish re-submit the manuscript entitled “**A genomic snapshot of *Salmonella enterica* serovar Typhi in Colombia**” for which I am the corresponding to be considered for publication as a research article in your journal. I confirm that: all contributing authors have seen and approved the manuscript, all authors have significantly contributed to the work and, that this article has not been published and or is being considered for publication elsewhere. Given that Typhoid from Latin America is regretfully neglected in most literature we think that PLoS Neglected Tropical Diseases is the ideal forum in which to communicate our findings.

Please find below the rebuttal to the reviewers comments.

Reviewer #1: No weak points in this manuscript. An important paper concerning the molecular epidemiology of S. Thypi. The paper concludes that the S. Typhi population structure in Colombia is mainly driven by endemic circulation of local genotypes. Interesting are the Colombian organisms in clades 2.5 and 3.5. These have their nearest neighbors isolated in India and Vietnam. International trade in agricultural, aquacultural and manufactured food products has facilitated the introduction of new Salmonella serovars within the geographical boundaries of importing countries. Is there perhaps a trade relation between these countries that explains the presence of these genotypes in Colombia? Colombia and Vietnam are the two major coffee export-oriented countries in the world. India and Colombia, as far as I know, have no trade agreements?

*We thank the reviewer for their comments.*

Reviewer #2: The manuscript titled “A genomic snapshot of Salmonella enterica serovar Typhi in Colombia” describes the inclusion and analysis of isolates of S. Typhi from Columbia. This manuscript adds new S. Typhi isolates from Colombia, a country on a continent where genome sequences of S. Typhi is largely absent, to the growing global collection of genome data. A technically-sound standardized characterization pipeline was followed using whole genome sequencing, SNP analysis, genotyping, and antimicrobial susceptibility testing. PFGE, in which the study team has extensive expertise, is used as a non-WGS comparator method and determined to be not adequate for the tracking of fine level analysis such as outbreaks and transmission events. While the study makes use of established pipelines and is well done, the primary hypothesis of this work is to address the lack of genomic information from Latin America and Columbia specifically. The data in this study is limited to Columbia, and there is no evidence that would suggest that the trends observed here are generally applicable to all Latin American countries. In fact, the first paragraph of the discussion would suggest that in fact the isolates from Columbia do not represent the rest of the Latin American isolates, even the small subset included there. The manuscript should be rewritten to address Columbia only and not attempt to extrapolate to all of Latin America.

*We thank the reviewer for their thorough comments and indeed agree that extrapolating this data set to the whole of Latin America might be not accurate. Via this paper we are aiming to seek collaborations to conduct a wider study in Latin America. We have moderated and removed the extrapolations to the whole of Latin America from the text in this manuscript.*

The finding that PFGE is not adequate for tracking transmission and outbreaks is not a new finding, but rather one of the reasons many have moved to WGS. This discussion can be removed from the study as it provides very little.

*We agree with the reviewer that this known; however, we think that this information is essential to highlight for a Latin American audience where PFGE is still the standard method to track outbreaks. We aimed to highlight the lack of concordance between PFGE and WGS again with a specific Latin American example with the hope that the Latin American typhoid community would begin to adopt WGS.*

Overall, the study is well done but the results and discussion should be reframed to focus on what can be said from this data. Additional comments on focusing the manuscript are included below.

*Agreed and this has been edited.*

Major comments:

The choice to perform a cross-sectional analysis of 77 (5.2%), rather than a comprehensive analysis of all or a majority of the 1,478 isolates from 1997 to 2018, is not well justified in light of the primary motivations of this manuscript, i.e., to fill in the gap/blind spot of S. Typhi genomics in South America, specifically in Colombia. In a previous publication by the authors [ref 18, doi:10.1371/journal.pntd.0008040], 402 isolates out of 846 reported typhoid cases from 2012-2015 were available for microbiological characterization and genotyping analyzed using PFGE, suggesting many available isolates were either not sequenced or not included. It is unclear why these isolates were not included. Under results (starting with line 184), the authors explain that isolate selections across the 22 years were made to maximize PFGE diversity, but later the authors conclude that PFGE correlated poorly with PFGE. Indeed, WGS of all available isolates could add an interesting

*We agree that sequencing the 1,500 Colombian isolates would be very interesting, however, there is currently no sequencing capacity in Colombia, and there were additional restrictions in the amount of DNA samples we were able to export from Colombia for sequencing. Therefore, we had to limit our ambitions to this subset of the available isolates, in order to suggest expanding sequencing capacity in Colombia and across Latin America.*

*In the subset we aimed to choose a diverse as possible subset covering samples from each year, distanced sample types and diverging PFGE patterns. Furthermore, as we were specifically interested to investigated whether the H58 clade could be detected in this country we selected most of the AMR presenting samples and a representative isolate from each of the known outbreaks. We have included the PFGE dendogram of all the 1,077 isolates and highlighted the selected isolates for better insight in the sample selection in Figure S1.*

Investigation into the clonality of the eight presumably distinct outbreaks from which single distinct PFGE isolates were selection (Fig. 1A), if it is fair to assume the outbreaks were designated as such due to a concordance of epidemiological linkage of persons, place, and time with a matching PFGE type.

*The outbreaks were defined by: Linkage of people and time with a matching PFGE pattern. However, as our aim was not to validate the outbreaks but to map the genetic diversity of genotypes present in Colombia and to detect if the H58 lineage were represented in the outbreaks The clonality of the outbreaks were only confirmed by WGS in 2 cases where more then 1 isolated from the outbreak was included for WGS.*

In another example where a lack of PFGE and genotype correlation is problematic for the study design, line 211 asserts a lack of genotype 4.3.1 (H58) in Colombia and line 2015 asserts limited accumulation of AMR genes,” but these statements may be false if one or several of the isolates excluded on the basis of PFGE pattern was in fact the H58 clone.

*We agree that we cannot (based on this small subset) conclude that H58 is not in Colombia. Nevertheless, taking into account that H58 is mainly associated with outbreaks, MDR and FQ -resistance, we included an isolate of each of the classical epidemiological confirmed outbreaks and most of the isolates that presented phenotypic AMR. These data imply that H58 between 1997 and 2018 was not a major genotype in Colombia (in other endemic areas H58 represents the majority of the sequenced isolates)*

Overall, this suggests to me that the inclusion criteria are flawed and should be reconsidered, or the implications of sampling bias should be further discussed. In its present form, the paper is an interim analysis that could be proven wrong by the same isolate collection on which it is based. Ideally, all available isolates would be sequenced and submitted to the public collection via this paper.

*Unfortunately, we do not have the capacity to sequence any further isolates and have discussed the implications of the sampling bias in more detail.*

If diverse PFGE patterns is of importance to the authors, a subsequent sub-analysis beginning with a cross-section of the maximal dataset could be suitable in order to reach the conclusion that PFGE does not correlate well with WGS genotype, which is one of the current genotyping standards in the field. Additional methodological detail on how the isolates were determined as the same or different should be included. While many readers will be familiar with this methodology, it is not clear in the current version of the manuscript (i.e. how many bands different were required to determine that two isolates were the same or different).

*We clarified the method section for PFGE typing to now read: “All organisms were subtyped by PFGE following standardized PulseNet protocols. [16]. Briefly, genomic DNA were digested with XbaI (Promega, USA) and run on gel. PFGE patterns from different gels were normalized by aligning the reference digestion pattern of S. Braenderup H9812.*

*The band assignation were assessed visually and by a computerized program (Gelcompare 4.0 software (Applied Maths, Belgium). Parameters of tolerance and optimization were set to 1.5% and similarities calculated according to Dice coefficient. The Clustering dendrogram was based on the unweighted pair-group method using arithmetic averages (UPGMA).*

*The resulting Resulting XbaI patterns were compared with the local database and if found indistinguishable (within this 1.5% tolerance) of an existing pattern the isolates was given the corresponding PFGE code, if a unique pattern was found a new PFGE code was assigned. All PFGE pattern codes were assigned following the PulseNet International guidelines for nomenclature, which includes 2 letters for the country or region, 3 letters for the serovar, 3 characters for the enzyme and 4 digits for the profile number (e.g. COINJPPX01.0001 for Colombia) [17]”.*

The authors suggest there are 60 unique PFGE patterns, but only 8 (A-H) are labelled in Figure 1A. The inclusion of the PFGE pattern in Figure 3, where most of the isolates appear to lack these designations is also confusing. Why are there no PFGE designations included.  Additionally Figure 1B does not add significantly to the submission and can be removed.

*Fig 1B has been removed as requested, In figure 1(previously 1A) and figure 3 the PFGE pattern is indicated by the PFGE code (there are 60 different codes included) In figure 1 A-H indicate isolates from classical epidemiological outbreaks. In figure 3 the PFGE patterns of the only the commonly found PFGE patterns is shown with the squares to easy visualisation of PFGE clustering along the WGS tree. The isolates with an unique PFGE pattern are not shown in squares, however all isolates have their PFGE code – from which the last numbers correspond to the PFGE pattern associated. We clarified this in the figure legend: “All the PFGE patterns are also indicated under PFGE code (the PFGE pattern correspond to the last digits of the PFGE code) as not all PFGE patterns where present more the once.”*

While the PFGE to WGS associations are interesting, they are not surprising considering the difference in the methodology.  However, with this dataset it would be good to interrogate the associations between the actual phenotypes and genotypes for AMR.

Line 74: missing the word “cases” after “13 million”

*This has been added.*

Lines 161-162 – what quality metrics were used to include or exclude any of the assembled genomes?  Any coverage, quality or contig size cutoffs were used in this study.

*Sanger pipelines were used - reference added- in line 174 and 182*

How were the AMR genes determined to be present or absent.  The current manuscript just indicates that they were screened, without any indication of what is considered present or absent.

*AMR genes were detected by SRST2 using the ARG-annot database using the standard cut offs. Text edited in line 191 to say: “standard cut-offs of >90% gene coverage and a minimum read dept of 5 reads were used.”*

Fig 2. Many colors for department are indistinguishable and appear to repeat. Consider recoloring, using numbers instead of colors, or adding a supplementary table with each isolate listed followed by key epidemiological and genotypic variables.

*Yes, we agree and a supplementary table has been added in S2*

Lines 206-9: commas to separate the list are missing, and capitalization of Column is inconsistent.

*These have been edited.*

Line 254: The source (presumably pathogen.watch) and the genome representation (alignment against CT18 or another local reference, or de novo assembly) of these 3,381 publicly available isolates would be helpful to know, as it is important that genomes are processed in the same way – aligned to CT18 or assembled – before being input into RAxML.

*All raw read sequences were processed identically i.e aligned to CT18 and SNP called using the sanger pipelines as described in M&M we clarified this by saying in line 180: “Sequenced reads and publicly available sequences were mapped and SNP called against the reference genome S. Typhi CT18 using the standard sanger pipelines and quality metrics as described in [26].”*

Lines 288-299: The suggestion that there is less AMR in many pathogens in Latina America is not supported by references or additional data and seems to be based on conjecture at this point.  These paragraph should be referenced appropriately or removed.

*The text has been adjusted to clarify that we meant less AMR in S. Typhi in Colombia*.

Line 418 – Data availability – it does not appear as though the data is publicly available with the included project ID.  The authors must ensure that all data, both raw sequences and assembled data are publicly available prior to resubmission as both are used in the analyses described in this study.

*These have been added to the ENA with the accession number*: *PRJEB42858*

The figure legends are lacking in any detail.

*We have added additional detail in the figure legends.*

Yours sincerely,

**Professor Stephen Baker BSc DIC PhD**

Wellcome Trust Senior Research Fellow

Director of Research for Global Health

Translation Microbiology and International Health
